# Atrioventricular Block in Celiac Disease: An Unusual Clinical Presentation in a Child. A Case-Based Review

**DOI:** 10.3390/children9111627

**Published:** 2022-10-26

**Authors:** Savina Mannarino, Sara Santacesaria, Irene Raso, Giulia Fini, Elena Pozzi, Cristina Cocuccio, Valeria Calcaterra, Gianvincenzo Zuccotti

**Affiliations:** 1 Pediatric Cardiology Department, “Vittore Buzzi” Children’s Hospital, 20154 Milan, Italy; 2 Pediatric Department, “Vittore Buzzi” Children’s Hospital, 20154 Milan, Italy; 3 Department of Internal Medicine, University of Pavia, 27100 Pavia, Italy; 4 Department of Biomedical and Clinical Science, University of Milano, 20157 Milano, Italy

**Keywords:** atrioventricular block, celiac disease, children, cardiac involvement, conduction disorders

## Abstract

Congenital or acquired atrioventricular block (AVB) is a rare disorder in the pediatric population, while celiac disease (CeD) is a common multisystemic autoimmune disorder that is characterized by intestinal manifestations as they are the typical clinical presentation. Sometimes CeD presents more complex multisystemic involvement which includes the heart. Cardiac involvement, such as dilated cardiomyopathy, myocarditis or conduction disease, have been mainly described in untreated adult patients with or without gastro-intestinal symptoms; rare cases of AVB and CeD have been also reported, particularly in association with extra-cardiac manifestations. We describe a case of a progressive acquired AVB block in a 4-year-old child, in which CeD was later diagnosed. A rapid and significantly improvement of the AVB grade has been obtained after the child started a strict gluten-free diet, and so we suggest including diagnostic exams for CeD in all of the children with acquired AVB.

## 1. Introduction

Atrioventricular block (AVB) is a rare conduction disorder in the pediatric population. It may occur in a structurally normal heart or in association with a congenital heart disease, and it can be congenital or acquired [1]. The acquired forms of it in children are secondary to surgical or catheterization-induced trauma, acute or chronic infection (such as Lyme disease, myocarditis), inflammatory processes (such as acute rheumatic fever, Kawasaki disease), electrolyte disturbances, medications, hypothyroidism, infiltrative processes (such as amyloidosis) [2], and immune-mediated injury of the conduction system which occurs as a result of transplacental passage of maternal anti-SSA/Ro-SSB/La antibodies [3].

Celiac disease (CeD) is a common multisystemic autoimmune disorder that is characterized by inflammation and villous atrophy in the small bowel, which are triggered by gluten ingestion in genetically predisposed individuals. The typical clinical presentation of CeD is characterized by intestinal manifestations such as diarrhea, a loss of appetite, abdominal distention, bloating, constipation, abdominal pain, and weight loss. Sometimes there are extra-intestinal symptoms: the most common ones are anemia, osteoporosis, fatigue, aphthous stomatitis, headache, and reproductive disorders [4,5,6,7]. The prevalence of extra-intestinal manifestations of CeD is similar between the pediatric patients and adults (approximately 60%), however the specific manifestations of it are different; the most common extra-intestinal CeD manifestations are a short stature in the pediatric population and iron deficiency anemia in adults [2,5,6,7].

Cardiac involvement (either idiopathic or immunological) has also been described in untreated adults or young adult patients with CeD [8]. Dilated cardiomyopathy, myocarditis [9], atrial fibrillation [10], and atherosclerosis with an increased risk of ischemic heart disease [11] are the most common manifestations that are reported in the literature. The association between CeD and conduction disorders such as AVB has been reported in very few clinical cases that concern only adults.

Furthermore, the data of cardiac improvement in patients who are treated with conventional therapy and a gluten-free diet (GFD) are inconclusive [9].

We report here a case of a 4-year-old girl with CeD who developed a high-grade AVB that significantly improved after she began a strict GFD. A literature review that focuses on the association between CeD and conduction disorders is also considered.

## 2. Case Presentation

**Figure 1:**
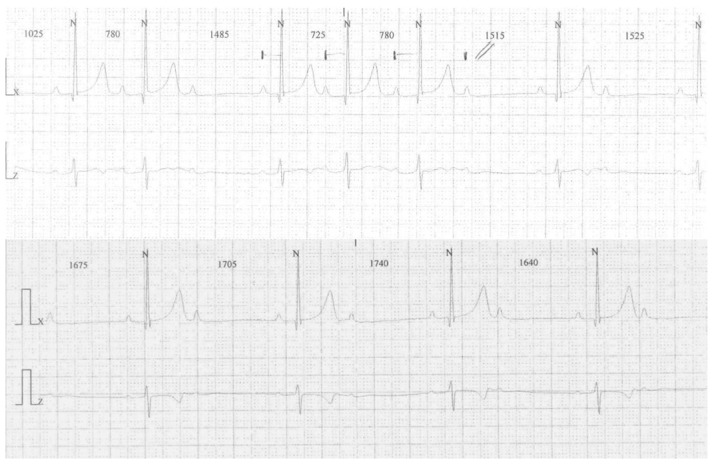
A 24h Holter monitoring (4 years) showing Mobitz type 1 atrio-ventricular block (AVB) (upper line) and 2:1 AVB (lower line).

A 4-year-old female (weight 16 kg, −0.09 z-score [12]; height 1.03 m, 0.36 z-score [12]; BMI 15.1 kg/m^2^, −0.34 z-score [12]) with neither a history of cardiac disease nor arrhythmia disorders in her family presented to our cardiology department for evidence of bradycardia during a routine pediatric evaluation. The 12-lead electrocardiogram (ECG) showed a 2:1 AVB, and the 24h Holter monitoring showed nocturnal phases of Mobitz type 1 AVB and episodes of 2:1 AVB (Figure 1).

No cardiac structural anomalies were found on the echocardiography. Ion disorders, previous or current infections, dysthyroidism, and anti-SSA/Ro and anti-SSB/La autoantibodies were also excluded by a blood sample test.

**Figure 2:**
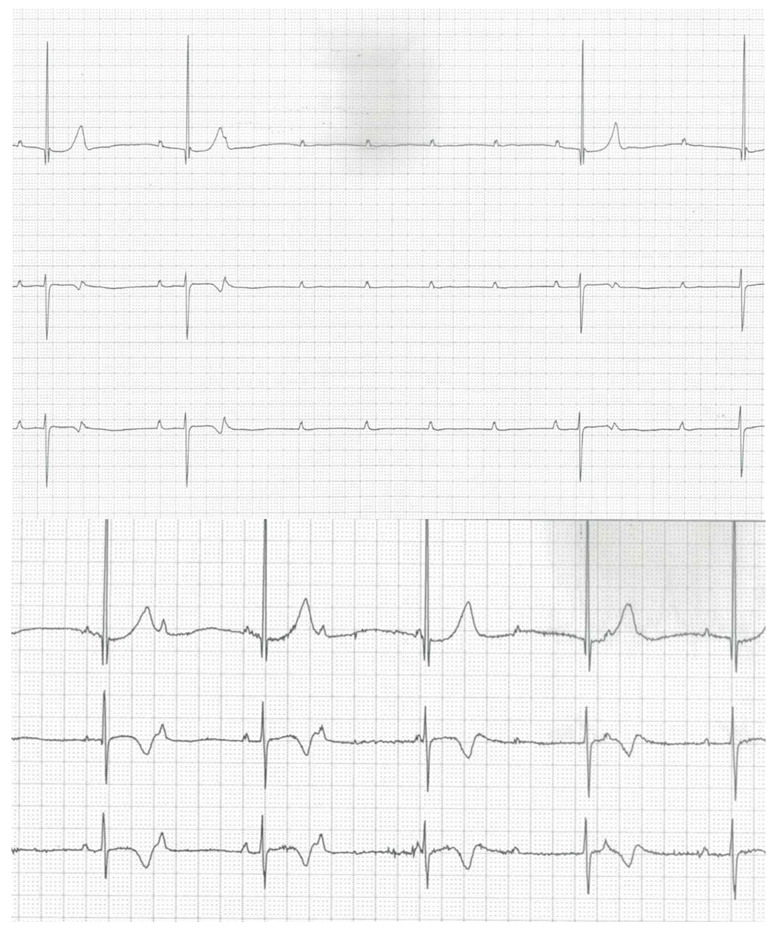
A 24 h Holter monitoring (8 years) showing a long nocturnal pause (upper line) and a daytime episode of III-degree atrio-ventricular block (lower line).

In the next four years, a worsening of the AVB was recorded up to III-degree AVB with a narrow QRS escape rhythm also in the daytime and pauses with the longest RR interval of 5.1 s (Figure 2). 

Chronotropic incompetence (latest maximum heart rate value 100 beats/min) and poor exercise capacity during the treadmill stress testing additionally appeared.

**Figure 3:**
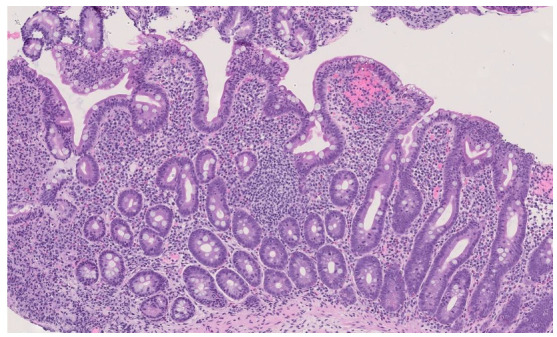
Duodenal mucosa showing partial villous atrophy and crypt hyperplasia with dense inflammatory infiltrate of the lamina propria and intraepithelial lymphocytes ILEs 42-44/100, H and E staining, original magnification 10×.

As the AVB worsened, the 8-year-old child experienced progressively more frequent episodes of headaches and abdominal pain. She had neither diarrhea nor a significant growth failure (weight 23 kg, −0.76 z-score [13]; height 1.29 m 0.30 z-score [13]; BMI 13.8 kg/m^2^, −1.39 z-score [13]). The blood tests detected normal levels of hemoglobin (14.1 g/dl, normal value > 11.5), iron (156 ug/dl, normal value: 60–130), ferritin (55 µg/mL, normal value: >100), folate (6.4 ng/mL, normal value: 2–9), vitamin B12 (1159 pg/mL, normal value: >400), TSH (1.03 µlU/mL, normal value 0.5–4.2), and only a low 25-OH-vitamin D level (18.7 ng/mL, normal value: >30). Despite her unspecific symptoms, she was investigated for gastrointestinal disorders due to her family history of inflammatory bowel disease (she has a father with Crohn’s disease). She was finally diagnosed with CeD because of the positivity of anti-transglutaminase antibodies, anti-endomysium antibodies (anti-tissue-transglutaminase IgA 25 Unit/milliliter, normal value: <5, anti-endomysium antibodies 1:160, normal value: negative), and mucosal changes in the duodenal biopsy. In Figure 3, the histological evaluation of the duodenal biopsy is shown.

**Figure 4:**
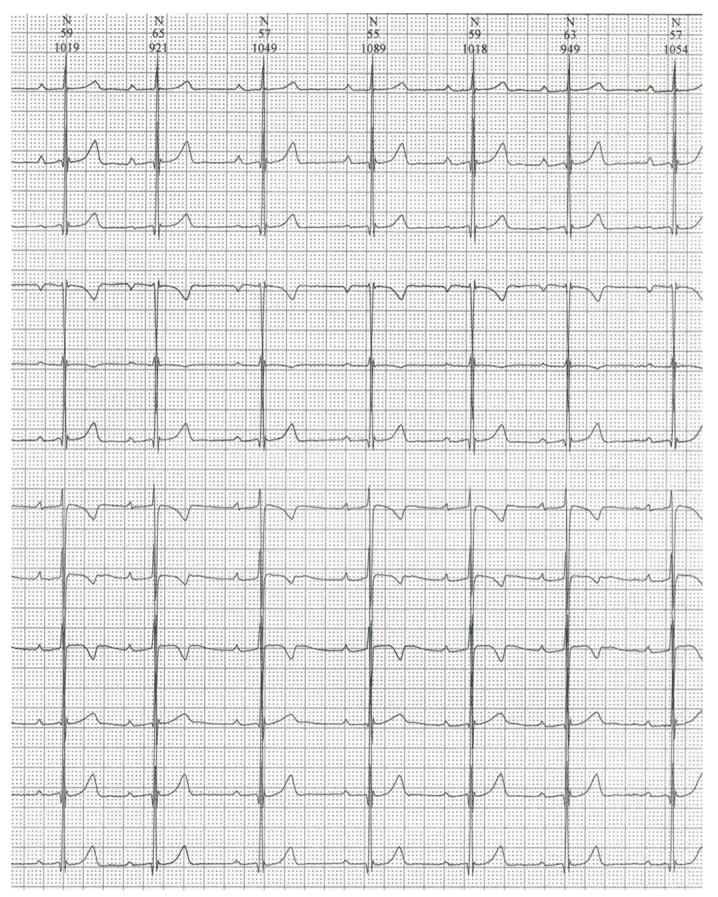
A 24h Holter monitoring (9 years) showing a I-degree atrio-ventricular block.

Immediately after the diagnosis, she started a strict GFD and after only 3 months, a remarkable improvement of the AVB was noticed. In the Holter monitoring, a regression of up to I-degree AVB was recorded with only brief episodes of Mobitz type 1 AVB during the nighttime (Figure 4). The finding remained stable, and it was confirmed over the next four years monitoring. The continuous negativity of the anti-transglutaminase antibodies at the blood exams confirmed the benefits of a strict GFD.

## 3. Discussion

CeD is a complex autoimmune condition that is characterized by a specific serological and histological profile, which is triggered by gluten ingestion in genetically predisposed individuals.

The estimated global prevalence of CeD is approximately 1%. CeD occurs at any age from early childhood to an adult age, with a first peak after gluten intake within the first 2 years of life and the second one in the second or third decade of life [4,5,6,7]. 

Its diagnosis remains a challenge due to its multifaceted clinical presentation and its variable age of recognition [14].

Classically, CeD is diagnosed among children presenting gastrointestinal manifestations of it, but a more complex multisystemic involvement can be observed with neurological, psychiatric, dermatologic, ocular, musculoskeletal, endocrine, pulmonary, and reproductive manifestations [15]. Moreover, in untreated CeD patients, a damaged cardiovascular system can be observed such as cardiomyopathy [16], myocarditis, pericardial effusion, arrhythmias, and premature atherosclerosis [17]. In some cases, young adults with auto-immune myocarditis, which is characterized by lymphocytic infiltrates, carry silent CeD that is usually not associated with any chronic gastrointestinal symptoms. In patients with myocarditis, CeD has an estimated prevalence between 1.8–5.7% which is markedly higher than it is in the general population [18]. Frustaci et al. reported a 4.4% incidence of CeD in patients with lymphocytic myocarditis. In this study, the nine described patients had iron deficiency anemia without gastrointestinal manifestations, and there was a substantial improvement of their cardiac symptoms after they started a GFD [9]. 

Additionally, a recent meta-analysis reported that adult patients having a later diagnosis of CeD, had a 38% increased risk of developing atrial fibrillation [10]. Rare cases of AVB with CeD in adult patients with an associated extra-cardiac manifestation have been reported [19,20,21,22,23] apart from a case of isolated AVB that was described in a 42-year-old white woman [17] presenting with syncope secondary to a high-grade heart block. In this patient, a CeD diagnosis was posed, and her conduction abnormalities promptly improved five months after a gluten-free diet introduction [17].

Interestingly, our case shows some peculiarities such as the onset of the AVB (4 years old) at a young age, the absence of any other cardiac or gastroenterological involvement at the time of the diagnosis, the appearance of new symptoms, such as abdominal pain and headaches as the AVB worsened. Furthermore, our patient had a significant history of autoimmune diseases in her family (she has a father with a severe form of Crohn’s disease). Acquired AVB is a rare condition in the pediatric age, and sometimes the treatment of the primary cause determines the improvement of the conduction disorder [24]. A challenging open question that is related to the cardiac involvement in CeD is the putative mechanisms of it and its reversibility. It is certain that patients with CeD are prone to developing additional autoimmune conditions during their life (such as Hashimoto’s thyroiditis, insulin-dependent diabetes mellitus, autoimmune hepatitis, and connective tissue disease). Furthermore, a family history of autoimmune disorders, as in our case, could make the patient genetically predisposed to them. The cardiac involvement underlies more possible mechanisms of it: a direct common autoimmune targeting the antigen which is present both in the myocardium and in the small intestine [25]; a myocardial damage that is caused by infectious agents that passed through the malfunctional intestinal barrier; the nutritional deficiency of minerals and vitamins that is caused by chronic malabsorption [8]. 

The risk of irreversible heart damage in unrecognized patients with CeD increases with age and with the duration of gluten exposure [16]. In our patient, the period of gluten exposure was not short (4 years), however, considering that all of the reported cardiac involvement in CeD appeared in adult people (after more than 20 years of gluten exposure in average) [26], the period of exposure in the present case can be considered to be limited, thus supporting the plausibility of heart derangement reversibility. In our studied child, no significant anemia or metabolic changes were present at the time of the diagnosis of CeD. However, we observed a direct causality between the diagnosis of CeD and the regression of the conduction disorder after the introduction of a strict GFD.

No large studies about the reversibility of cardiac events after gluten avoidance are present in the literature, and the data are discordant. Some cases report a complete or partial recovery of the cardiac involvement, while others are unresponsive to the diet [8]. In our case, an early diagnosis of the bowel disease, together with a strict treatment for it, may have promoted the significant improvement of the conduction disorder.

## 4. Conclusions

We presented an unusual case of an association between AVB and CeD. To the best of our knowledge, this is the first reported case in the pediatric population. Even if the data on the association between CeD and conduction disorders are rare, the hypothesis of an autoimmune reaction involving both the myocardium and the small intestine cannot be excluded. The current case is important since it provides an important insight to the clinical presentation of CD in patients of pediatric age as extra-intestinal manifestations of the cardiac involvement could be not excluded. In the children with acquired AVB who return negative results for the tests for other more frequent pathological conditions, diagnostic CeD exams should be also considered.

## Data Availability

The data presented in this study are available on request from the corresponding author. The data are not publicly available due to privacy reasons.
